# CREB1-BCL2 drives mitochondrial resilience in RAS GAP-dependent breast cancer chemoresistance

**DOI:** 10.1038/s41388-025-03284-5

**Published:** 2025-01-31

**Authors:** Ki-Fong Man, Omeed Darweesh, Jinghui Hong, Alexandra Thompson, Charlotte O’Connor, Chiara Bonaldo, Mark N. Melkonyan, Mo Sun, Rajnikant Patel, Leif W. Ellisen, Tim Robinson, Dong Song, Siang-Boon Koh

**Affiliations:** 1 University of Bristol, University Walk, Bristol, UK; 2 College of Pharmacy, Al-Kitab University, Kirkuk, Iraq; 3 Department of Breast Surgery, General Surgery Centre, The First Hospital of Jilin University, Changchun, Jilin China; 4 Massachusetts General Hospital, Boston, MA USA; 5 Department of Pathology, The First Hospital of Jilin University, Changchun, Jilin China; 6 University of Leicester, Henry Wellcome Building, Lancaster, UK; 7 Harvard Medical School, Boston, MA USA; 8 University Hospitals Bristol and Weston, NHS Foundation Trust, Bristol, UK

**Keywords:** Oncogenes, Breast cancer, Apoptosis

## Abstract

Triple-negative breast cancer (TNBC) is an aggressive and heterogenous breast cancer subtype. RASAL2 is a RAS GTPase-activating protein (GAP) that has been associated with platinum resistance in TNBC, but the underlying mechanism is unknown. Here, we show that RASAL2 is enriched following neoadjuvant chemotherapy in TNBC patients. This enrichment is specific to the tumour compartment compared to adjacent normal tissues, suggesting that RASAL2 upregulation is tumour-selective. Analyses based on 2D/3D cultures and patient-derived xenograft models reveal that RASAL2 confers cross-resistance to common DNA-damaging chemotherapies other than platinum. Mechanistically, we found that apoptotic signalling is significantly downregulated upon RASAL2 expression. This feature is characterised by substantial alterations in the expression of anti-versus pro-apoptotic factors, pointing to heterogeneous mechanisms. In particular, RASAL2 upregulates BCL2 via activation of the oncogenic transcription co-factor YAP. CREB1, a YAP-interacting protein, was identified as the common transcription factor that binds to the promoter regions of RASAL2 and BCL2, driving their collective expression. A subset of RASAL2 colocalises with BCL2 subcellularly. Both proteins decorate mitochondria, where the high levels of mitochondrial RASAL2-induced BCL2 expression render the organelles refractory to apoptosis. Accordingly, mitochondrial outer membrane permeabilisation assay using live mitochondria from RASAL2-high/chemoresistant tumour cells demonstrated attenuated release of death signal, cytochrome c, when exposed to pro-apoptotic factors BAX and tBID. Similarly, these cells were more resilient towards chemotherapy-induced mitochondrial depolarisation. Together, this work reveals a previously undocumented molecular link between RAS GAP and apoptosis regulation, providing a new mechanistic framework for targeting a subset of chemorefractory tumours.

## Introduction

Chemoresistance is a clinical challenge, and among the diverse mechanisms, deregulation of the RAS pathway is an established driver of chemoresistance in many tumour contexts [1, 2]. Classically, RAS deregulation is mediated by genetic mutations of the RAS gene. More recently, direct RAS regulators––namely, guanine nucleotide exchange factors (GEFs) and GTPase-activating proteins (GAPs)––have emerged as mediators through which nongenetic RAS deregulation is orchestrated. Much of the phenotypic and functional implications of these regulators is unknown [3].

Defined by an absence of oestrogen and progesterone hormone receptors as well as a lack of HER2 receptor tyrosine kinase amplification, triple-negative breast cancer (TNBC) has the worst prognosis of all breast cancer subtypes with higher rates of relapse and shorter overall survival. TNBC tumours are more likely to harbour high genomic instability and deficiency in homologous recombination repair that predicts sensitivity to DNA-damaging agents, including platinum and topoisomerase inhibitors [4]. Nonetheless, these standard chemotherapeutic agents are often associated with low response rates, owing to the substantial biological heterogeneity of the disease [5]. For example, unlike in hormone receptor-positive breast cancers, a failure to achieve complete eradication of viable tumour cells following chemotherapy (clinically defined as pathologic complete response) in TNBC is associated with worse outcomes in terms of cancer relapse. This association is consistent with the notion that minor subpopulations of TNBC cells are responsible for and capable of driving treatment failure, cancer progression and ultimately disease relapse. Therefore, understanding the biology of therapy-resistant TNBC represents a key step towards addressing the unmet needs of TNBC patients [6, 7].

RASAL2 is a canonical RAS-GAP, i.e., it promotes the hydrolysis of RAS-GTP to RAS-GDP thereby inactivating RAS. As a RAS-GAP, RASAL2 is a known tumour suppressor in a number of cancer types. For example, ablation of RASAL2 leads to tumour proliferation and metastasis in hormone receptor-positive breast tumours, as a result of RAS hyperactivation [8]. Paradoxically, RASAL2 is also an established oncogene in aggressive cancer types such as TNBC, pancreatic ductal adenocarcinoma, colorectal cancer and gastric cancer [9–11]. In these contexts, RASAL2 has been found to have tumorigenic functions outside of its RAS regulatory role. For example, in TNBC, RASAL2 physically associates with small GTPase RAC-GAP, activating the RAC1 axis and leading to metastatic progression [9]. In gastric cancer, RASAL2 binds to protein phosphatase 2 A, leading to oncogenic activation of β catenin signalling [10].

In keeping with the oncogenic propensity of RASAL2 in TNBC, we previously reported our observation that RASAL2 was overexpressed in the pre-treatment TNBC tumours of patients who had the worst treatment outcome following platinum-based chemotherapy [12, 13]. While these and other studies have begun to clarify the oncogenic implications of RASAL2, its molecular mechanism in mediating therapeutic responses in any cancer contexts remains undefined. Here, we sought to elucidate the molecular mechanism through which RASAL2 promotes chemoresistance in TNBC. We found that RASAL2 is a tumour-specific factor in residual TNBC and is capable of driving resistance towards a broad range of common chemotherapeutic agents beyond platinum. Molecular analyses using preclinical experimental models and patient specimens revealed that this phenotype is orchestrated by a hitherto undescribed interaction between RASAL2 and apoptosis factors, where there was a concerted suppression of pro-apoptotic activity upon RASAL2 overexpression, leading to apoptotic tolerance. Together, our findings offer new insights into the mechanistic role of RASAL2 in dictating cellular fate in response to treatment.

## Results

### Residual post-treatment TNBC tumours are enriched with RASAL2 compared to adjacent normal breast tissues

**Fig. 1:**
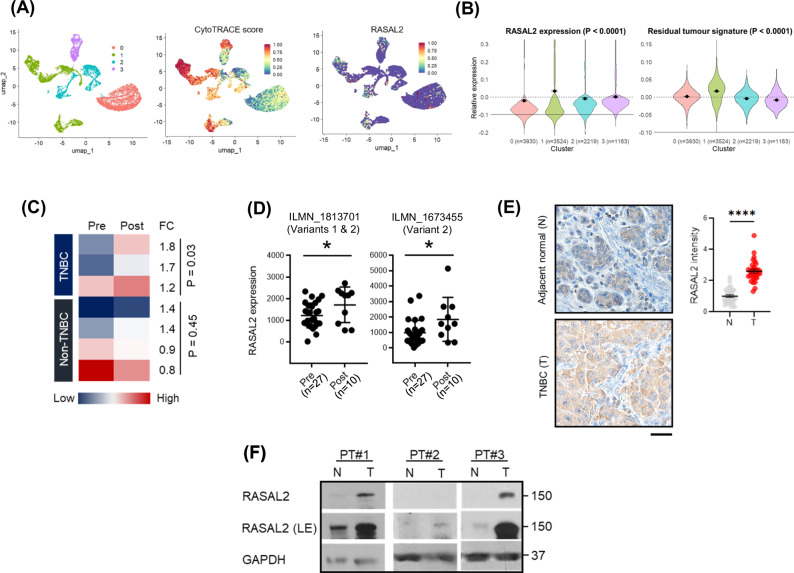
Residual tumours, but not adjacent normal tissues, are enriched with RASAL2. **A** UMAP plots of epithelial cells from primary TNBC tumours showing correlation between CytoTRACE score (1—least differentiation, 0 – most differentiation) and RASAL2 expression. **B** Violin plots of RASAL2 expression and residual tumour signature expression in the four clusters of epithelial cells identified in the TNBC tumours in (**A**). Cluster 1 expressions are significantly higher compared to all other clusters. Data are represented as mean ± SEM. *P* value by one-way ANOVA. **C** Heatmap of RASAL2 expression in pre- and post-treatment breast cancer patients [19]. Fold change (FC) was determined relative to pre-treatment expression level. *P* value by paired *T*-test. **D** Dot plots of RASAL2 expression in pre- versus post-treatment TNBC/*BRCA*-mutant breast cancer patients [20]. Probes that recognise RASAL2 variant 2 (ILMN_1813701 and ILMN_1673455) show significant increase following treatment. Data are represented as mean ± SEM. *P* value by two-tailed *T*-test. **E** Immunohistochemistry of fixed post-treatment TNBC patient breast specimens. RASAL2 (brown stain) was enriched in the tumour compartment versus adjacent normal epithelia. Data are represented as mean ± SEM. *P* value by two-tailed *T*-test. Scale bar, 20 µm. **F** Immunoblotting of fresh post-treatment TNBC patient specimens. RASAL2 was enriched in the tumour (T) versus adjacent normal (N) tissues in TNBC patients. LE long exposure.

Undifferentiated tumours are associated with poorer prognosis compared to well-differentiated tumours [14]. Using CytoTRACE, a computational framework for predicting differentiation states from single-cell RNA-sequencing data [15, 16], we found that high RASAL2 expression overlapped with less differentiated epithelial cells from TNBC patients (Fig. 1A). Moreover, high RASAL2 expression in these cells was strongly associated with a tumour transcriptomic signature [17] derived from genes enriched in residual viable tumour population of patients treated with pre-operative chemotherapy (Fig. 1B). Further validation using another cohort of TNBC patients [18] showed that RASAL2 was specifically enriched in the most aggressive subpopulation that harbour multiple signatures related to unfavourable clinical outcomes such as metastasis and chemoresistance (Fig. S1A). Collectively, despite the heterogeneity of TNBC, these data suggest that RASAL2 is a common molecular feature in a subset of aggressive TNBC cells.

Next, we asked how RASAL2 expression changed post-treatment. First, we analysed the transcriptome of patient-matched breast tumour specimens before and after neoadjuvant (pre-operative) chemotherapy [19]. Consistently, RASAL2 was upregulated following treatment in tumours of TNBC patients, but this upregulation was not observed in some of the non-TNBC patients (Fig. 1C). There are at least two known RASAL2 transcriptional variants, variants 1 and 2, where they differ in the 5’ untranslated and coding regions (Fig. S1B). Using an independent patient cohort of TNBC/*BRCA*-mutant breast cancer patients [20], we found that transcripts of RASAL2 variant 2, but not variant 1, was significantly upregulated following treatment (Figs. 1D and S1C, D). Furthermore, we observed that RASAL2 protein was enriched in post-treatment (residual) TNBC tumour tissue compared to patient-matched adjacent normal breast tissue (Fig. 1E). Immunoblotting of fresh patient-matched residual tumour versus normal breast tissues corroborated this observation (Figs. 1F and S1E). Collectively, these findings suggest that RASAL2 is a tumour cell-specific factor that is upregulated in residual treatment-resistant TNBC.

### RASAL2 promotes pan-resistance to cytotoxic agents beyond platinum

**Fig. 2:**
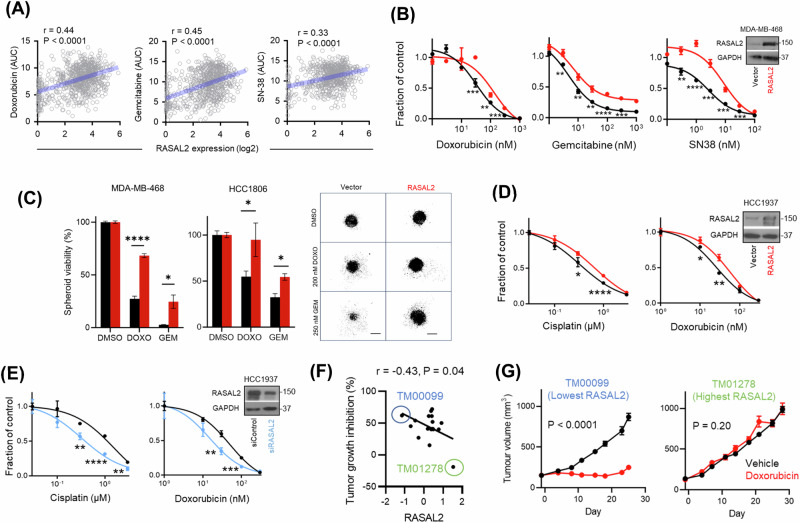
RASAL2 promotes pan-resistance to cytotoxic agents beyond platinum. **A** Correlation between RASAL2 expression and sensitivity to indicated chemotherapy. The area under percent-viability curves (AUC) was computed as a metric of drug sensitivity, as derived from the Cancer Therapeutics Response Portal. Pearson *r* and *P* value are reported. **B** Cell viability assay. Vector control and RASAL2-overexpressing MDA-MB-468 cells were treated as indicated. Data are represented as mean ± SEM, *n* = 3 biological replicates. *P* value by paired *T*-test. **C** Spheroid assay. Viability of vector control and RASAL2-overexpressing TNBC spheroids was measured following treatment with vehicle DMSO, doxorubicin (DOXO) or gemcitabine (GEM). Representative images of HCC1806 spheroids are shown. Data are represented as mean ± SEM, *n* = 3 biological replicates. *P* value by paired *T*-test. Scale bar, 250 µm. Cell viability assay. Vector control and RASAL2-overexpressing (**D**) or -knockdown (**E**) HCC1937 cells were treated as indicated. Data are represented as mean ± SEM, *n* = 3 biological replicates. *P* value by paired *T*-test. **F** Correlation between RASAL2 expression and in vivo tumour response to doxorubicin. Mice were treated with vehicle control or 2 mg/kg doxorubicin [22]. Each dot represents an independent TNBC PDX model. Tumour growth inhibition was defined as [1 − (mean volume of treated tumours)/(mean volume of control tumours)] × 100%. Pearson *r* and *P* value are reported. **G** Change in tumour volume following doxorubicin in two TNBC PDX models. Mice were treated as described in (**F**), *n* = 8–9 per group. TM00099 tumours had the lowest RASAL2 expression, whereas TM01278 had the highest RASAL2 expression. *P* value by two-way ANOVA test.

We previously reported that platinum resistance is correlated with RASAL2 overexpression [12, 13], but the extent of this correlation with other DNA-damaging agents was largely unexplored. Using the publicly available computational analysis of resistance model [21], we found that RASAL2 expression is highly associated with resistance towards drugs that target the DNA damage checkpoint-related pathways (Fig. S2A). Pharmacogenomic analysis of human cancer cell lines revealed that RASAL2 was broadly associated with resistance to classic DNA-damaging agents (Fig. S2B). Among others, these agents include chemotherapeutic agents that are used in TNBC such as doxorubicin, gemcitabine and SN-38 (payload of FDA-approved antibody-drug conjugate sacituzumab govitecan) (Fig. 2A). In-vitro cytotoxic assays using 2D TNBC cell lines and 3D tumour spheroids confirmed the association. Ectopic expression of RASAL2 was sufficient to render cells more resistant to these drugs (Figs. 2B–D and S2C, D). Conversely, knockdown of endogenous RASAL2 reversed the resistance phenotype (Figs. 2E and S2D). *BRCA1*-mutant TNBC cells exhibited similar chemosensitivity profiles following RASAL2 overexpression and knockdown (Fig. 2D, E), suggesting that RASAL2-driven chemoresponse is at least in part independent of wild-type BRCA1 function. In a cohort of established TNBC patient-derived xenograft (PDX) mice [22], again, RASAL2 expression is significantly correlated with chemoresponsiveness (Fig. 2F). The PDX model with the highest expression of RASAL2 was doxorubicin-resistant, whereas the model with the lowest expression was highly sensitive (Fig. 2G). Together, these findings show that the effect of RASAL2 in driving resistance is not limited to platinum-based therapy as previously shown, but also to other common DNA-damaging cytotoxic agents.

### BCL2 is upregulated by RASAL2

**Fig. 3:**
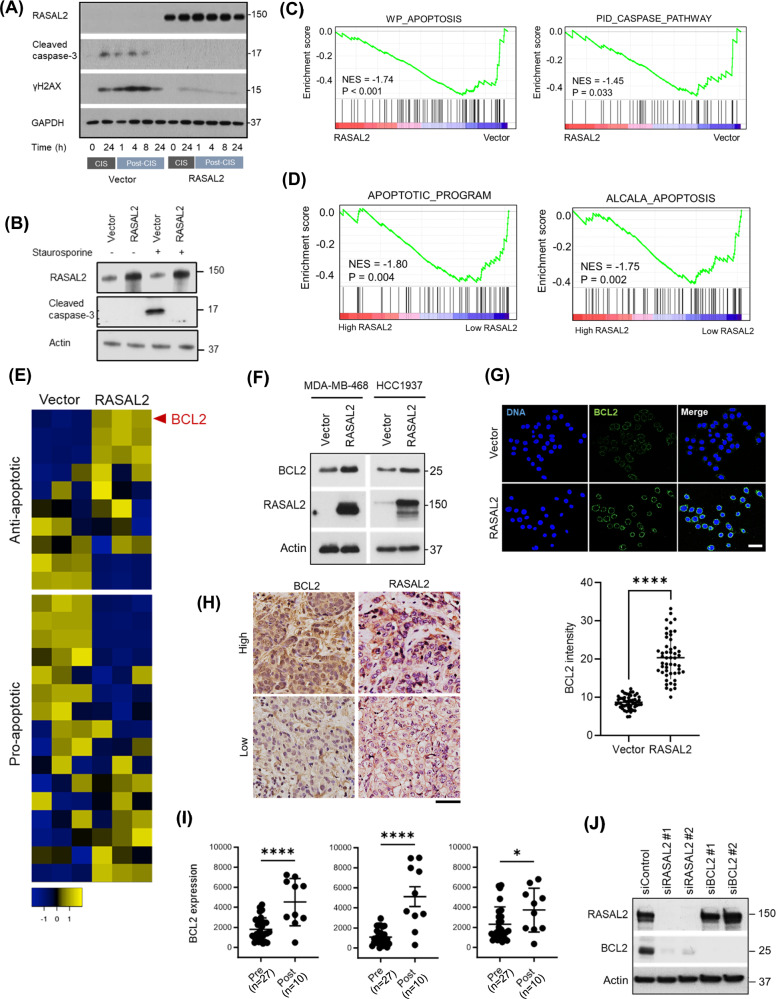
RASAL2 upregulates BCL2. **A** Immunoblotting of cisplatin-treated TNBC cells showing upregulation of apoptotic/DNA damage markers, cleaved caspase 3 and γH2AX, respectively. Vector control and RASAL2-overexpressing MDA-MB-468 cells were treated with 3 µM cisplatin for 24 h before being released into fresh drug-free medium for the indicated durations. **B** Immunoblotting showing upregulation of apoptotic marker, cleaved caspase 3, in vector control but not RASAL2-overexpressing MDA-MB-468 cells following apoptosis induction. Cells were treated with either DMSO or 10 µM staurosporine for 1 h. **C** GSEA analysis of transcriptomes showing downregulation of apoptosis signatures in RASAL2-overexpressing MDA-MB-468 cells compared to isogenic vector cells. **D** GSEA analysis of transcriptomes showing downregulation of apoptosis signatures in RASAL2-high TNBC tumours versus RASAL2-low TNBC tumours. TNBC patients from the TCGA cohort were stratified by quartile of RASAL2 expression. **E** Heatmaps of apoptosis-related genes for isogenic RASAL2-overexpressing MDA-MB-468 cells. BCL2 was the topmost significantly upregulated anti-apoptotic gene in RASAL2-overexpressing cells. **F** Immunoblotting of isogenic TNBC cell lines showing upregulation of BCL2 protein upon RASAL2 expression. **G** Quantitative immunofluorescence of BCL2 expression in isogenic RASAL2-overexpressing MDA-MB-468 cell model. Data are represented as mean ± SEM. *P* value by two-tailed *T*-test. Scale bar, 40 µm. **H** Representative immunohistochemistry showing TNBC tumours with high RASAL2/BCL2 protein expression (top panel) versus low RASAL2/BCL2 protein expression (bottom panel). Quantification was done based on the H-score system (Fig. S3E). Scale bar, 50 µm. **I** Dot plots showing upregulation of BCL2 mRNA expression in post-treatment primary TNBC/*BRCA*-mutant tumours versus pre-treatment tumours. Data are represented as mean ± SEM. *P* value by two-tailed *T*-test. **J** Immunoblotting of isogenic MDA-MB-468 cells following siRNA knockdown of RASAL2 or BCL2. Knocking down RASAL2 downregulated BCL2 expression, while knocking down BCL2 did not change RASAL2 expression.

We observed that chemotherapy-induced apoptosis by 24 h far more robustly in control TNBC cells compared to RASAL2-overexpressing TNBC cells, as evidenced by concerted upregulation of both ɣH2AX and cleaved caspase 3 (Fig. 3A). Using a classic apoptosis inducer staurosporine, we found that RASAL2 expression indeed rendered cells less susceptible to apoptosis (Fig. 3B). To determine the underlying mechanisms, we performed genome-wide RNA sequencing of the isogenic RASAL2-overexpressing TNBC MDA-MB-468 cell model. Gene set enrichment analysis (GSEA) revealed downregulation of apoptotic transcriptomic signatures in RASAL2-overexpressing cells (Figs. 3C and S3A), in line with the prediction that RASAL2 is a resistance gene against apoptotic signals (Fig. S2A). In The Cancer Genome Atlas (TCGA) clinical cohort [23], TNBC tumours with high RASAL2 expression exhibited attenuated apoptotic signalling compared to those with low RASAL2 expression (Fig. 3D). This clinical correlation was also observed in another independent cohort of TNBC patients [24] (Fig. S3B). We found that anti-apoptotic genes such as BCL2, BCL-XL and BAXI1 were significantly upregulated upon RASAL2 expression, whereas pro-apoptotic genes such as BIK, BIM and BOK were significantly downregulated (Fig. S3C). By ranking all anti-apoptotic genes based on fold change, BCL2 emerged as the topmost elevated factor in RASAL2-overexpressing cells (Fig. 3E). We asked whether this transcriptional upregulation was recapitulated at the protein level. Immunoblotting of TNBC cell lines revealed that BCL2 protein was increased following RASAL2 expression (Fig. 3F). To further support these findings, we performed quantitative immunofluorescence and found that BCL2 protein level was significantly higher in RASAL2-overexpressing cells compared to control cells (Fig. 3G).

We asked whether the association between RASAL2 and BCL2 expression was clinically relevant. The trend of a positive correlation between BCL2 and RASAL2 was observed at the mRNA and protein level in our own TNBC clinical specimens (Figs. 3H and S3D, E). In independent clinical cohorts, where RASAL2 was elevated in TNBC tumours post-treatment (Fig. 1C, D), BCL2 was also significantly elevated post-treatment (Figs. 3I and S3F). BCL2 and RASAL2 are targets of transcription co-factor YAP [12, 25, 26], which itself is activated by RASAL2 via the inactivation of the Hippo pathway [11]. In concordance, we found that overexpressing RASAL2-induced YAP activation, while silencing RASAL2 decreased YAP activation and increased translocation of YAP from the nucleus to cytoplasm (Fig. S3G–I). Accordingly, overexpressing RASAL2 upregulated BCL2 (Fig. 3F–G), while knocking down RASAL2 downregulated BCL2 (Fig. 3J). On the other hand, knocking down BCL2 did not lead to a change in RASAL2 expression (Fig. 3J), suggesting that BCL2 acts downstream of RASAL2. Finally, knocking down YAP reversed the upregulation of BCL2 by RASAL2 (Fig. S3J). Together, these findings suggest that RASAL2 upregulates BCL2 via YAP, and that this axis contributes to the concerted dampening of apoptotic signalling observed in the RASAL2-high context.

### CREB1 is the common transcription factor that drives BCL2 and RASAL2 expression

**Fig. 4:**
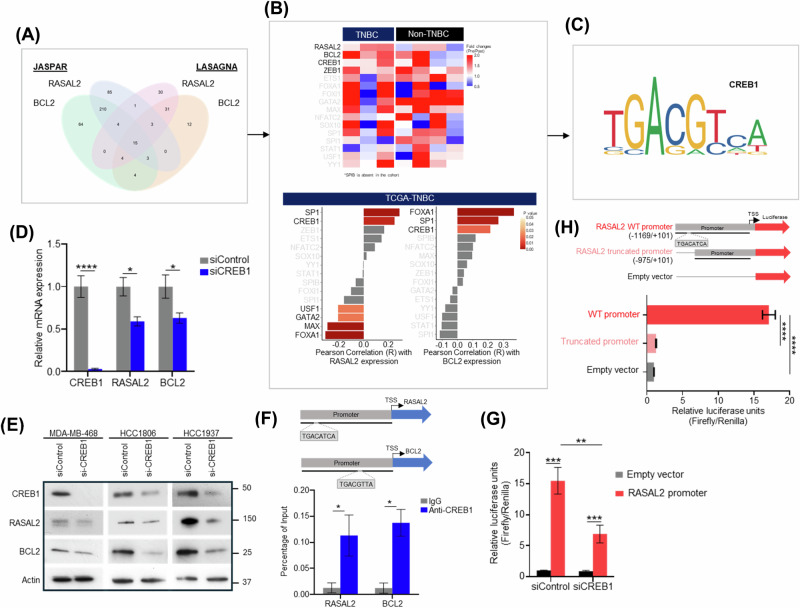
Transcription factor CREB1 drives RASAL2 and BCL2 expression. **A** Venn diagram showing the overlapped predicted transcription factors binding on the promoters of RASAL2 and BCL2 using computational tools, JASPAR and LASAGNA. **B** Analyses of candidate transcription factors in breast cancer patient cohorts. Heatmap shows the fold changes of RASAL2, BCL2 and candidate gene transcription factors in patient-matched breast tumour specimens post- versus pre-treatment (top, [19]). Correlation between RASAL2/BCL2 expression and candidate transcription factors in TNBC patients in the TCGA-BRCA cohort (bottom). *P* value by two-sided Pearson correlation analysis. **C** Consensus binding motifs of transcription factor CREB1. **D** Decrease in the relative mRNA expression of RASAL2 and BCL2 following siRNA-mediated knockdown of CREB1 in MDA-MB-468 cells. Data are represented as mean ± SEM, *n* = 3 biological replicates. *P* value by two-tailed *T*-test. **E** Immunoblotting of TNBC cells transfected with siCREB1 or control siRNA. CREB1, RASAL2 and BCL2 were decreased in expression in cells treated with siCREB1 compared to control. **F** ChIP-qPCR confirmation of CREB1 binding to predicted sites on RASAL2 and BCL2 promoters. TSS denotes transcription start site. Data are represented as mean ± SEM, n = 3 biological replicates. *P* value by two-tailed *T*-test. **G** Decrease in the relative luciferase units in siCREB1 MDA-MB-468 cells compared to siControl. pRL-CMV Renilla luciferase plasmid was co-transfected for normalisation. Data are represented as mean ± SEM, *n* = 3 biological replicates. *P* value by two-way ANOVA. **H** Decrease in the relative luciferase units in MDA-MB-468 cells with truncated RASAL2 promoter without CREB1-binding sequence compared to those with wild-type (WT) RASAL2 promoter. pRL-CMV Renilla luciferase plasmid was co-transfected for normalisation. Data are represented as mean ± SEM, *n* = 3 biological replicates. *P* value by one-way ANOVA test.

The collective YAP-mediated upregulation of BCL2 and RASAL2 suggested that there might be common transcription factors driving their expression. To determine the upstream regulatory elements, we first employed computational tools JASPAR (a repository of transcription factor binding profiles represented as position frequency matrices [27]) and LASAGNA-Search 2.0 (a database leveraging the Length-Aware Site Alignment Guided by Nucleotide Association algorithm for identifying transcription factor binding sites [28, 29]) to predict transcription factors that most likely bind to the promoters of RASAL2 and BCL2. Our convergent analysis identified 15 candidate transcription factors for both genes (Fig. 4A). We filtered this list by analysing transcriptomics data from patient-matched breast tumour specimens collected before and after neoadjuvant (pre-operative) chemotherapy. We found that, similar to RASAL2 and BCL2, two of the 15 transcription factors, CREB1 and ZEB1, were consistently upregulated in post-treatment samples specifically in TNBC but not in non-TNBC patients [19] (Fig. 4B, top). In particular, CREB1 is a YAP-interacting transcription factor [30] that was associated with not only post-treatment upregulation [19, 20] (Figs. 4B and S4A, B) but was also predictive of poor outcomes in chemotherapy-treated TNBC patients [31] (Fig. S4C). Furthermore, correlative analysis using TNBC patients in the TCGA-BRCA cohort showed that CREB1 demonstrated a significant positive correlation with both RASAL2 and BCL2 expressions (Fig. 4B, bottom).

Given that CREB1 is the sole candidate transcription factor that demonstrated notable clinical correlations across multiple patient cohorts (Figs. 4B, C and S4A–C), we proceeded to test the functional effect of knocking down CREB1. First, we found a decrease in both mRNA and protein expression levels of RASAL2 and BCL2 upon CREB1 knockdown across all TNBC cell lines tested (Fig. 4D, E and S4D). Second, we assessed the binding of CREB1 to the respective promoters of RASAL2 and BCL2 by ChIP-qPCR. At predicted sites within RASAL2 and BCL2 promoter regions, we found significant enrichment of CREB1 binding (Fig. 4F). Furthermore, CREB1 ChIP-sequencing data demonstrated a significant binding peak at the predicted CREB1 binding site in the RASAL2 promoter region [32], indicating a strong likelihood that CREB1 interacts with the RASAL2 promoter (Fig. S4E). Third, we generated RASAL2 promoter and performed luciferase reporter assay. We observed a significant reduction in luciferase activity when CREB1 was silenced in TNBC cells (Fig. 4G), suggesting that CREB1 is responsible to enabling RASAL2 transcription. Finally, we truncated RASAL2 promoter region harbouring the binding sequence of CREB1, and again found a significant reduction in luciferase activity compared to wild-type promoter (Fig. 4H). Together, these findings show that CREB1 is a transcription factor that is, at least in part, responsible for the upregulation of RASAL2 and BCL2.

### BCL2 interacts with RASAL2 on mitochondria

**Fig. 5:**
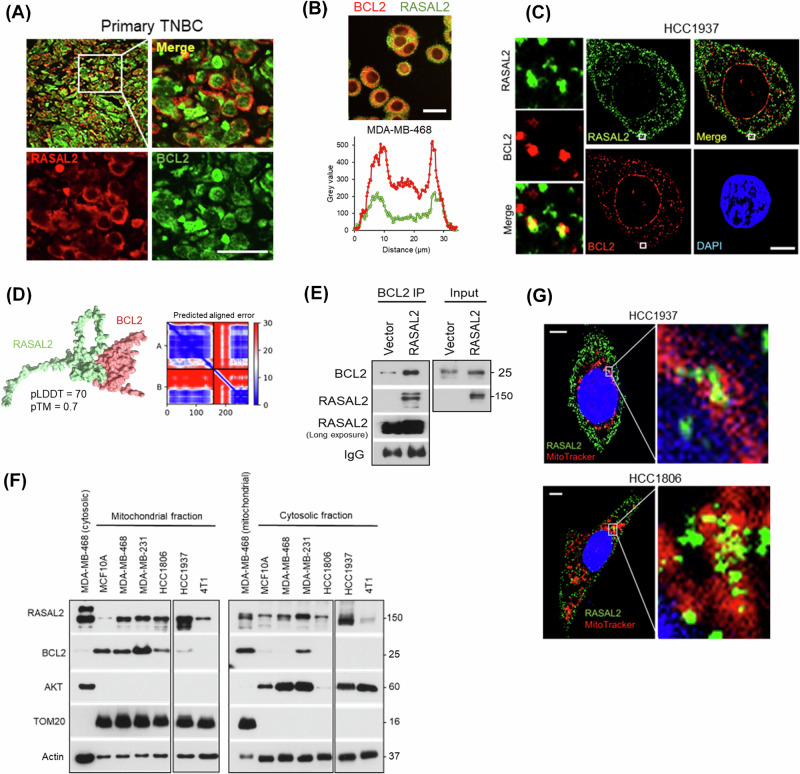
Mitochondria is a common homing site for BCL2 and RASAL2. **A** Immunofluorescence of BCL2 and RASAL2 in primary TNBC patient tumour. Scale bar, 20 µm. **B** Immunofluorescence of BCL2 and RASAL2 in TNBC cells. Bottom graph shows the line scan quantification of BCL2 (red) and RASAL2 (green). Scale bar, 30 µm. **C** Confocal imaging of BCL2 and RASAL2 in TNBC cells. Panels on the left show exemplary co-localisation of signals within the boxed region of the cell. Scale bar, 10 µm. **D** AlphaFold prediction of the interaction between BCL2 and the N-terminus of RASAL2. pLDDT score (0–100) is a confidence score, and pTM score (0–1) is a metric for the structural congruency between two folded protein structures, with higher scores corresponding to higher confidence. PAE plot of the top ranked model is shown on the right [33, 34]. **E** Co-immunoprecipitation of BCL2 and RASAL2 in MDA-MB-468 cells. **F** Immunoblotting of cytoplasmic versus mitochondrial fractions of mammary cell lines. BCL2 was not detected in 4T1 murine cells as the antibody used was reactive only to human. AKT and TOM20 serve as cytoplasmic and mitochondrial markers, respectively. **G** Confocal imaging of RASAL2 and MitoTracker in TNBC cells. Scale bar, 5 µm.

In TNBC cells and patient tumours, we observed that immunofluorescence staining of BCL2 overlapped with RASAL2, suggesting subcellular co-localisation of the two proteins (Fig. 5A, B). To validate these findings, we employed high-resolution confocal microscopy and found that BCL2 colocalised with a subset of RASAL2 (Figs. 5C and S5A). In-silico AlphaFold modelling predicted high confidence protein-protein interaction between BCL2 and the N-terminus of RASAL2 [33, 34] (Fig. 5D), which was consistent with the result of co-immunoprecipitation assay (Fig. 5E). Moreover, unlike the full-length wild-type RASAL2, truncation of the N-terminus of RASAL2 abolished the ability of RASAL2 to bind to BCL2 (Fig. S5B). Given that BCL2 normally localises to mitochondria, we hypothesised that a subset of RASAL2 may be present on these organelles. Subcellular fractionation of multiple mammary cell lines showed that the detection of BCL2 was largely restricted to the mitochondrial fraction, as expected (Fig. 5F). Conversely, RASAL2 was found in both the cytosolic and mitochondrial fractions. Indeed, the mitochondrial presence of RASAL2 was detected in all cell lines used, including non-malignant human mammary line MCF10A and malignant mouse mammary line 4T1, alluding to an evolutionarily conserved feature (Fig. 5F). Mitochondrial labelling in cells further demonstrated an overlap of RASAL2 with the mitochondrial network, corroborating the notion of co-localisation (Figs. 5G and S5C). Collectively, these findings reveal a hitherto undescribed physical interaction between BCL2 and RASAL2, with mitochondria being a common homing site for both proteins.

### High BCL2 levels confer mitochondrial resilience against apoptosis induction

**Fig. 6:**
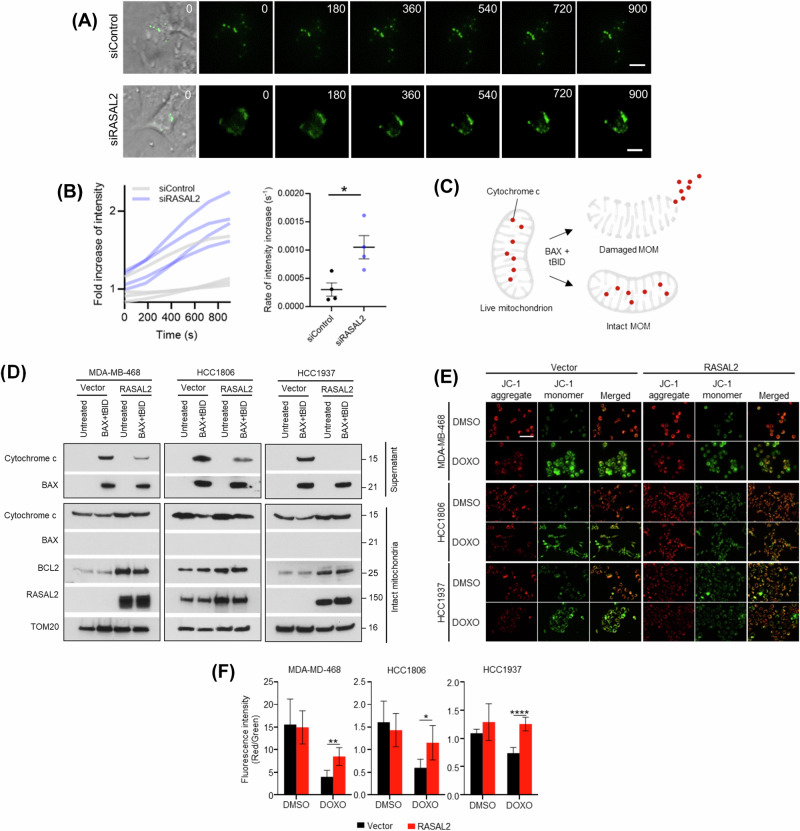
BCL2 upregulation attenuate mitochondrial depolarisation by attenuating BAX oligomerisation. **A** Live-cell imaging. RASAL2 depletion increases the rate of GFP-BAX accumulation (green) in HCC1937 cells following exposure to 20 µM staurosporine. Number denotes time in seconds. Scale bar, 10 µm. **B** Quantification of change in BAX intensity. Fluorescence intensity of individual BAX foci was tracked over time and quantified, *n* = 4 foci per condition. Data are represented as mean ± SEM. *P* value by two-tailed *T*-test. **C** Schematic for live mitochondrial outer membrane permeabilisation (MOMP) assay. **D** MOMP assays revealing attenuated cytochrome c release in RASAL2-overexpressing TNBC cells. TOM20 serves as mitochondrial marker. **E** JC-1 mitochondrial membrane potential assay. TNBC cells were treated with vehicle DMSO or 5 µM doxorubicin (DOXO), and subsequently stained with JC-1 reagent. JC-1 aggregates (indicating high mitochondrial membrane potential) were observed as red, while JC-1 monomers (indicating low mitochondrial membrane potential) were green. Representative images of vector control and RASAL2-overexpressing TNBC cells are shown. Scale bar, 100 µm. **F** Quantification of the ratio of integrated intensity of red to green fluorescence in (**E**). Data are represented as mean ± SEM, *n* = 5 random fields of view per condition. *P* value by two-tailed *T*-test.

BAX translocates to mitochondria and oligomerises in response to apoptotic stimuli, leading to permeabilisation of mitochondrial outer membrane [35, 36]. We hypothesised that increased BCL2 levels in the RASAL2-high context counteract BAX oligomerisation and render mitochondria more robust to permeabilisation. We conducted live-cell imaging of TNBC cells transfected with GFP-tagged BAX. As expected, upon exposure to staurosporine, BAX fluorescence intensity increased over time, consistent with its accumulation following apoptosis induction (Fig. 6A). The kinetics of BAX accumulation in RASAL2-depleted cells was faster than that in control cells, indicating that RASAL2 attenuates BAX accumulation during apoptosis induction (Fig. 6B).

A hallmark of apoptosis is the disruption of the mitochondrial outer membrane. To directly test the resilience of mitochondria, we isolated live mitochondria from TNBC cells and measured the level of death signal cytochrome c following apoptosis induction (Fig. 6C). First, consistent with the higher levels of total BCL2 found in RASAL2-high cells (Fig. 3F, G), mitochondria of these cells harbour more BCL2 compared to the isogenic control cells (first versus third columns, Fig. 6D). Second, in line with the presence of RASAL2 in mitochondrial fraction (Fig. 5F), RASAL2 was also detected on intact mitochondria. In fact, similar to BCL2, mitochondrial RASAL2 level was higher in RASAL2-high cells compared to control cells (first two versus last two columns, Fig. 6D). Third, following apoptosis induction by treatment of BAX and tBID, release of cytochrome c was detected in the supernatant. Notably, there was less cytochrome c detected in the supernatant of RASAL2-high mitochondria compared to control cells, supporting the notion that RASAL2-high cells are more apoptosis-tolerant (second versus fourth columns, Fig. 6D). The same observations were made across different TNBC cell lines (Fig. 6D). Finally, to further confirm that RASAL2 bolsters mitochondrial resilience to depolarisation, we used JC-1 dye as a live-cell mitochondrial membrane potential indicator in TNBC cells exposed to chemotherapy (Fig. 6E). At high membrane potentials, JC-1 dye monomer forms aggregates, where it accumulates within the mitochondria. Conversely, at low internal mitochondrial concentrations or low membrane potentials, the dye presents as monomers. Consistent with the findings of the mitochondrial outer membrane permeabilisation assay (Fig. 6C, D), we found significantly more JC-1 aggregates (red) in RASAL2-overexpressing cells compared to control cells upon doxorubicin treatment (Fig. 6E, F), indicating a higher mitochondrial tolerance towards apoptosis induction in the former. This observation was uniformly recapitulated across multiple cell lines and DNA-damaging agents (Figs. 6E, F and S6A, B). Furthermore, consistent with the notion that CREB1 drives RASAL2 expression (Fig. 4), we found significantly reduced accumulation of JC-1 aggregates (red) in CREB1-knockdown cells compared to control cells following chemotherapy treatment (Fig. S6C, D), suggesting a lower mitochondrial tolerance to apoptosis in the absence of CREB1. Together, these findings suggest that RASAL2 bolsters mitochondrial resilience by mitigating membrane depolarisation, presumably via inhibition of BAX oligomerisation owing to high levels of mitochondrial BCL2.

## Discussion

The biological heterogeneity of TNBC is underpinned by multiple pro-tumour mechanisms that contribute to the survival of the tumour cells [37]. Previously, we found that RASAL2 is a key determinant of platinum resistance in TNBC [12, 13]. Here, we established that in fact the RAS GAP confers pan-resistance to a range of DNA-damaging agents, including payload of antibody-drug conjugates sacituzumab govitecan and trastuzumab deruxtecan, which are used in patients of TNBC and other cancer types. We further showed that RASAL2 upregulates the expression of anti-apoptotic BCL2 family members, collectively leading to a dampening of apoptotic signalling. Among these anti-apoptotic factors, RASAL2-induced elevation of BCL2 expression at mitochondria enhances mitochondrial resilience against depolarisation, as evidenced by attenuation of cytochrome c release and diminished oligomerisation of pro-apoptotic BAX upon apoptotic stimuli. Consequently, RASAL2-high tumour cells are able to withstand chemotherapy-induced apoptosis, effectively evading cell death. Consistent with its role in driving chemoresistance, in patients, RASAL2 is enriched in residual tumours following neoadjuvant chemotherapy. This enrichment is notably absent in adjacent non-malignant tissues, suggesting that targeting its associated pathways is likely to be tumour-specific.

**Fig. 7:**
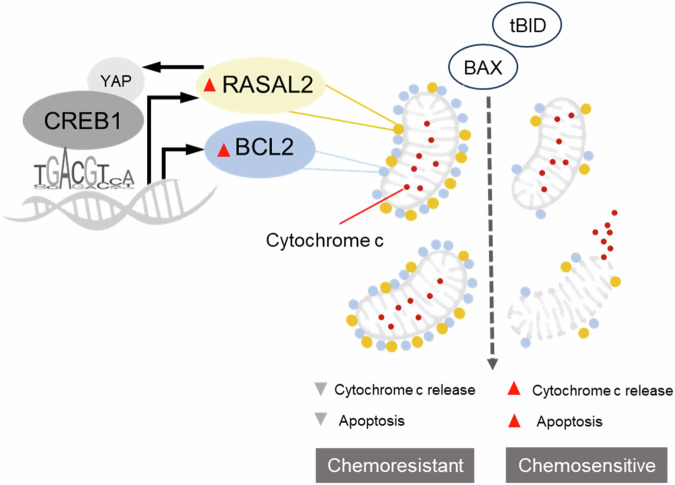
Mechanism of apoptotic regulation by the CREB1-RASAL2-BCL2 axis. RASAL2 and BCL2 share common transcription factor motifs in their promoter regions. Transcription factor CREB1 binds to these promoter regions, and drives the expression of RASAL2 and BCL2. This upregulation is supported by CREB1-interactor YAP, a transcription co-factor that is regulated by RASAL2, thus forming a positive loop in the CREB1-RASAL2-BCL2 axis. Both RASAL2 and BCL2 colocalise at the mitochondria. Their presence confers mitochondrial resilience by mitigating mitochondrial outer membrane depolarisation, which occurs, for example, during BAX/tBID-triggered apoptosis. Consequently, in high RASAL2/BCL2 chemoresistant tumour cells, there is reduced cytochrome c release upon apoptosis induction and thereby attenuation of cell death.

Emerging studies have uncovered unanticipated protein–protein interactions for direct RAS regulators such as RASAL2. For example, RASAL2 has been found to bind to RAC-GAP ARHGAP24, leading to activation of the RAC1 pathway that contributes to TNBC metastasis [9]. In colorectal cancer, RASAL2 interacts with Hippo regulator LATS2, preventing YAP from degradative ubiquitination and thereby enabling its oncogenic function as a nuclear transcription co-factor [11]. In gastric cancer, RASAL2 has been found to be physically associated with protein phosphatase PP2A, leading to oncogenic activation of the β catenin signalling [10]. In this work, we have established physical association between RASAL2 and BCL2 at the mitochondria, where RASAL2-driven upregulation of BCL2 confers mitochondrial resilience. We further showed that the collective upregulation of RASAL2 and BCL2 is driven by YAP-interactor CREB1, a transcription factor whose binding motif corresponds to specific sites within RASAL2 and BCL2 promoters. Our proposed model of mechanism (Fig. 7) reveals a hitherto undocumented crosstalk between RAS GAP and BCL2 regulation mediated via YAP-CREB1 transcription factor complex. This crosstalk enables tumour cells to be more resistant towards apoptosis induction, underpinning chemoresistance.

Evasion of apoptosis is an established hallmark of cancer. Our work provides evidence of a direct modulation of the apoptosis pathway by a RAS GAP. While RAS is canonically known to inhibit apoptosis, differential effects of RAS on this pathway have been described [38]. Specifically, mitochondrial RAS has been shown to execute pro-apoptotic effects in eukaryotes. For example, studies using budding yeast models have shown that activated Ras2 (one of the mammalian RAS homologues) induces mitochondrial dysfunction, accumulation of reactive oxygen species and apoptosis [39–41]. In mammalian cells, translocation of KRAS from plasma membrane to mitochondria is orchestrated by protein kinase C, which triggers apoptosis [42]. Another study also found that RAS potentiates apoptotic signalling at the mitochondria in a manner that is dependent on the prenylation of its C-terminal CAAX motif [43]. Our findings showed that RASAL2 not only upregulates anti-apoptotic factors such as BCL2 but also localises to the mitochondrial compartment. Thus, in addition to dampening apoptotic signalling, it is conceivable that being a RAS GAP RASAL2 may further bolster the anti-apoptotic ability of tumour cells by directly attenuating mitochondrial RAS pro-apoptotic signalling. It remains to be elucidated whether there are other RAS-independent functional roles of mitochondrial RASAL2.

In sum, this work has elucidated a role of the CREB1-RASAL2-BCL2 axis in mediating resistance to a broad range of DNA-damaging agents, through suppression of the apoptotic pathway. Given the multiple targetable factors involved in this network [44–46], our data illustrate a distinct mechanistic perspective that could serve as a reference for future work on overcoming the associated chemoresistance.

## Materials and methods

### Cell lines and chemicals

All cell lines were obtained from either the European Collection of Cell Cultures or the ATCC. They were grown in cell culture medium (RPMI or DMEM) supplemented with 10% v/v FBS (Sigma) as well as 1% v/v penicillin/streptomycin (Gibco), and were used up to a maximum of 25 passages following thawing. Mycoplasma testing was routinely performed using MycoAlert Mycoplasma Detection Kit (Lonza) prior to freezing. Cisplatin and carboplatin were dissolved in 0.9% w/v sodium chloride, while all other drugs (doxorubicin, gemcitabine, SN38, staurosporine) were dissolved in DMSO. Final DMSO concentrations were kept constant (≤0.2%) in all experiments.

### Lentiviral transduction and siRNA transfection

For lentiviral transduction, HEK293T cells were transfected while at 70% confluency with pLenti CMV Puro DEST (w118-1; Addgene #17452) or R777-E235 Hs.RASAL2 (Addgene #70519) together with lentiviral packaging plasmids using the CalPhos Mammalian Transfection Kit (Clontech Laboratories). Conditioned medium containing lentiviral particles were collected after overnight transfection and were filtered with 0.45-μm pore filter (Millipore). Filtered media were then used to transduce target cells, with addition of polybrene (Sigma) at a final concentration of 10 μg/mL. The transduced cells were selected using 1 μg/μL puromycin (Sigma) for a week and were maintained in medium with 1 μg/μL puromycin.

For siRNA transfection, human-specific siRNA (ThermoFisher Scientific) was transfected into cells by Lipofectamine RNAiMAX transfection reagent (ThermoFisher Scientific) according to manufacturer’s instructions. After 72 h, the transfected cells were collected for further analysis. The full list of primers are in Supplementary Table 1.

### Cell viability assays

Cells were seeded 24 h in 96-well plates before treatment exposure for specified duration. Cell viability was measured by CellTiter-Glo Luminescent Assay (Promega) according to manufacturer’s instructions. Alternatively, cells were fixed with 70% v/v methanol and stained with 0.1% crystal violet. Crystal violet was solubilised using 10% v/v acetic acid and the absorbance was measured at 590 nm.

### Spheroid generation and analyses

Cells were seeded on ultra-low attachment 96-well U-shaped-bottom microplate (ThermoFisher Scientific). After 72 h, when spheroid formation had established, drug treatment was performed for another 96 h. Cellular ATP level was measured with CellTiter-Glo 2.0 Cell Viability Assay (Promega). Images were recorded using a phase contrast microscope (Nikon).

### Patient sample collection

Breast tumour and adjacent normal breast specimens from TNBC patients were used in this study, with appropriate ethical approval and informed consent [12, 47, 48]. For post-treatment samples, they were initially sent for standard histological processing and evaluation. Once it was confirmed that there were adequate tissues for clinical use, part of the leftover tissues was released for research. Specimens were collected either in saline solution or 4% v/v buffered formalin for downstream processing and analyses.

### RNA sequencing and analysis

Total RNA was extracted using the RNeasy Mini Kit (QIAGEN) according to the manufacturer’s instructions. All samples had an RNA integrity number (RIN) of 10. Library was prepared from total RNA using the PolyA selection protocol. Libraries were sequenced using the HiSeq sequencing platform (Illumina) under the conditions of a 2 × 150 bp configuration, resulting in approximately 350 million paired-end (PE) sequencing reads per lane. The data quality met the requirement of at least 80% of bases having a minimum quality score of Q30. Sequencing reads were filtered for adaptor and low-quality sequences. Data were processed for quality control, alignment to the Human Genome GRCh38, quantification of gene expression, and differential expression analysis using standard bioinformatics pipelines.

### RNA extraction, cDNA synthesis and quantitative PCR

Total RNA was extracted by RNeasy Mini Kit (QIAGEN) and cDNA was synthesised with LunaScript® RT SuperMix Kit (New England Biolabs) according to manufacturer’s instructions. qPCR was performed using QuantiNova SYBR Green PCR Kit (QIAGEN) on a MxPro Mx3005P qPCR system (Agilent Technologies) with data analysed by Mx Pro qPCR software (Agilent Technologies). Relative expression differences were calculated using the 2^−∆∆Ct^ method. The full list of primers and related details are in Supplementary Table 1.

### Immunoblotting

Cells were seeded in 6-well plates at 250,000–500,000 cells per well and, following the indicated treatment, were lysed in RIPA buffer (50 mmol/L Tris pH 8, 150 mmol/L sodium chloride, 5 mmol/L EDTA, 0.5% w/v sodium deoxycholate, 0.1% w/v sodium dodecyl sulfate, 1% v/v NP-40, as well as protease and phosphatase inhibitors). Protein concentrations were quantified by the Bio-Rad Protein Assay (Bio-Rad). Equal amounts of protein were resolved using the SDS–PAGE system and transferred to polyvinylidene difluoride membranes (Millipore). Membranes were blocked with 5% w/v milk in TBST and incubated with primary antibodies overnight (4 °C) followed by horseradish peroxidase (HRP)-conjugated secondary antibodies for 1 h (room temperature). The signal was detected by enhanced chemiluminescence (PerkinElmer) and X-ray film. The full list of antibodies and related details are in Supplementary Table 2.

### Co-immunoprecipitation

Proteins from cell lysates were immunoprecipitated using Pierce protein A/G magnetic beads. The magnetic beads (25 µL/sample) were harvested with a magnetic stand and washed with ice-cold washing buffer. The cell lysate (1–1.5 mg protein) was precleared with 25 µL protein A/G magnetic beads according to the manufacture’s protocol. The precleared cell lysate was incubated with 10 µL (2 µg) antibody for 3–4 h at 4 °C on a rotator. The immunocomplexes were captured by adding prewashed magnetic beads (50 µL) and incubated for an additional 3–4 h at room temperature or overnight at 4 °C with mixing. The magnetic beads were then collected and washed with ice-cold washing buffer. The beads were re-suspended in 25 µL freshly prepared 2× SDS-PAGE sample buffer for 20 min at room temperature, to elute the immunoprecipitated proteins from the complex. The beads were sedimented with the magnetic stand, and the supernatant was collected, and analysed (without heating at 100 °C) by immunoblotting. The full list of antibodies and related details are in Supplementary Table 2.

### Chromatin immunoprecipitation (ChIP) assay

ChIP was performed with the Magna ChIP® A/G Chromatin Immunoprecipitation Kit (Millipore). In brief, cells were crosslinked by 1% v/v formaldehyde in PBS. DNA was then sonicated and immunoprecipitated with anti-CREB1 (Cell Signaling) or rabbit IgG control (Sigma). The immunoprecipitated and eluted DNA was purified with columns and amplified by qPCR with the following primers: CREB1-RASAL2—(F: 5′-TGGTGAAACTCTCCTTGGGTG-3′), (R: 5′-TCTTTGTGCAGTGTGTTGGC-3′) and CREB1-BCL2— (F: 5′-TCAGAGGAGGGCTCTTTCTTT-3′), (R: 5′-CATTCTCTGCACAGCCCGAC-3′).

### Immunofluorescence and microscopy

Cells were grown on sterile glass coverslips in 6-well plates. Cells were fixed in ice-cold methanol for 30 min at -20 °C or 3.7% v/v formaldehyde at room temperature for 10 min, followed by permeabilisation with 0.1% v/v Triton X-100 for 10 min, where necessary. Fixed cells were then washed with PBS prior to incubation with 1% w/v bovine serum albumin in PBS containing 0.1% v/v Tween-20 (PBST) for 45–60 min at room temperature. This step was followed by incubation with the appropriate primary antibodies for 1 h at room temperature, or overnight at 4 °C. After washing, the cells were incubated with the appropriate secondary antibody for 1 h at room temperature in the dark before visualisation. Confocal images were captured using the Leica TCS SP5 confocal laser scanning microscope. Images were analysed using ImageJ software. The full list of antibodies and related details are in Supplementary Table 3.

### Immunohistochemistry and scoring of specimens

Tissues were fixed in 4% v/v buffered formalin and kept in 70% ethanol until paraffin embedding. Paraffin sections of tissues were dewaxed and hydrated with alcohol. The sections were incubated with 3% peroxidase for 15 min at room temperature, followed by incubation with the primary and secondary antibodies of choice for 60 min at 37 °C. Slides were evaluated by the H-score system. Briefly, random fields of view from each section were selected, where the intensity of staining and the percentage of positive cells were the two core parameters to measure. Staining intensity was defined as negative (0 points), weakly positive (1 point), moderately positive (2 points), and strongly positive (3 points). The final score was the sum of the staining intensity multiplied by the average percentage of positive cells in the field of view. Therefore, the H-score ranges from 0 to 300. The H-score assessment was done independently by two pathologists in a blinded manner. The full list of antibodies and related details are in Supplementary Table 3.

### Luciferase reporter assay

The full-length and truncated RASAL2 promoters were amplified from the TNBC cell line MDA-MB-468 and cloned into the pGL3-basic luciferase reporter vector (Promega) using the primers (F: 5′-AAAACGCGTACTATGTCCAAGGCACGGTT-3′ and F: 5′-AAAACGCGTAGCCAACACACTGCACAAAG-3′, respectively; R: 5′-AAAGCTAGCAGAGGACGCAGAAGAACCCG-3′) with PrimeSTAR GXL DNA Polymerase (TAKARA). The luciferase reporter constructs, pRL-CMV Renilla luciferase control and siRNA were transiently co-transfected into the TNBC cell line MDA-MB-468 using Lipofectamine 2000 reagent (Thermofisher Scientific). Luciferase activity was determined using the Dual-Glo Luciferase Assay System (Promega) and detected by the GloMax Explorer System (Promega). Luciferase reporter activity was represented as a ratio of firefly to Renilla luminescence.

### Generation and immunoprecipitation of RASAL2 variants

To generate full-length wild-type and N-terminally truncated RASAL2 variants, RASAL2 was amplified from MDA-MB-468 and cloned into the XLG3-EGFP vector, which was kindly provided by Dr. Borko Amulic (Bristol), using primers (F: AAAGTCGACGGCACCATGGAGCTCTCT-3′ and F: 5′-AAAGTCGACCCTAATAAGGACAATTGCAGGCGA-3′, respectively; R: 5′-TTTACGCGTTTAGCAGCTGCTGTTTTTGAATTCACC-3′) and PrimeSTAR GXL DNA Polymerase (TAKARA). The RASAL2 variants were transfected into MDA-MB-468 cells using Lipofectamine 2000 reagent (Thermofisher Scientific). The full-length wild-type RASAL2 and the N-terminal truncated (lacking nucleotides +1 to +819) variants from cell lysates were then immunoprecipitated using GFP-Trap® agarose beads (ChromoTek). 25 µL of agarose beads per sample were washed with ice-cold washing buffer and then incubated with 1 mg of precleared cell lysate at 4 °C overnight on a rotator. The beads were then collected by centrifugation, washed with ice-cold washing buffer, and re-suspended in 80 µL of freshly prepared 2× SDS-PAGE sample buffer. Samples were heated at 100 °C for 10 min, and the supernatant was collected for subsequent analysis by immunoblotting. The full list of antibodies and related details are in Supplementary Table 2.

### Live-cell imaging

Fluorescence-based live-cell imaging assay was performed as previously described [49–51]. Briefly, cells were seeded onto chamber slides (ibidi) under cell culture conditions for 24 h. Time-lapse images were taken using Leica DMI6000 inverted epifluorescence microscope. Images were analysed using ImageJ software.

### Live mitochondrial outer membrane permeabilisation (MOMP) assay

Cells were harvested and centrifuged at 1000 G for 5 min at 4 °C. The cell pellet was re-suspended (in two-thirds of the volume of the cell pellet) in cold mitochondrial isolation buffer (250 mM sucrose, 10 mM HEPES pH 7.4) supplemented with protease inhibitors, and incubated for 10 min on ice. The cells were homogenised by 20 strokes with a 1-mL syringe Braun 25 G (0.5 mm × 16 mm cannula). The homogenate was centrifuged at 800 G for 5 min at 4 °C to remove nuclei and unbroken cells, and supernatant containing heavy membrane fractions and cytosol was collected. The supernatant was then centrifuged at 10,000 G for 20 min at 4 °C to pellet the mitochondrial fraction. The supernatant was transferred to a new Eppendorf tube and subsequently subjected to a centrifugation step at 150,000 G for 30 min at 4 °C (cytosolic fraction). Isolated live mitochondria were then split into two sets. The first set of mitochondria were incubated with recombinant wild-type BAX (100 nM) in association with recombinant tBID (Bio-Techne, 10 nM) for 1 h at 37 °C. Mitochondria were incubated with the recombinant proteins in 200 μl of KCl buffer (125 mM KCl, 4 mM MgCl_2_, 5 mM Na_2_HPO_4_, 5 mM succinate, 0.5 mM EGTA, 15 mM HEPES–KOH at pH 7.4, 5 μM rotenone). As control, the second set of mitochondria were incubated with the same buffer without the recombinant proteins for 1 h at 37 °C. Finally, the samples were centrifuged at 13,000 G for 5 min at 4 °C, and the resultant mitochondrial pellets and supernatant were resolved by SDS–PAGE. The degree of cytochrome c release into the supernatant and the level of cytochrome c remaining in the mitochondrial pellets were assessed by immunoblotting with anti-cytochrome c (BD Pharmingen).

### Measurement of live-cell mitochondrial membrane potential

TNBC cells were seeded in chamber slides (ibidi) and treated with chemotherapeutic agents on the following day. After 3 or 4-h treatment, the intracellular mitochondrial membrane potential levels were measured using the JC-1 Mitochondrial Membrane Potential Detection Kit (Biotium) according to the manufacturer’s instructions. Briefly, cells were incubated at 37 °C for 15 min with 1× JC-1 reagent working solution, and then washed with medium. The JC-1 stained live cells were imaged using the Celldiscoverer 7 (ZEISS). Fluorescence was measured at excitation/emission wavelengths of 550 nm/600 nm for red fluorescence and 485 nm/535 nm for green fluorescence. Images were analysed as the ratio of red fluorescence to green fluorescence using ImageJ software.

### Statistical analyses

Data were analysed using the GraphPad Prism or Microsoft Excel. Unless otherwise denoted, comparison between two groups was performed using Student *t*-test, unpaired or paired. Comparison among three or more groups were performed using either one-way or two-way ANOVA test, followed by post hoc Tukey multiple comparisons test. The following asterisk rating system for *P* value was used: *P < 0.05; **P < 0.01; ***P < 0.001; ****P < 0.0001.

## Supplementary information

Supplementary figures

Supplementary tables

## Data Availability

All relevant data generated in this study are available within the article and supplementary files.
