# Correction: CREB1-BCL2 drives mitochondrial resilience in RAS GAP-dependent breast cancer chemoresistance

**DOI:** 10.1038/s41388-025-03369-1

**Published:** 2025-04-02

**Authors:** Ki-Fong Man, Omeed Darweesh, Jinghui Hong, Alexandra Thompson, Charlotte O’Connor, Chiara Bonaldo, Mark N. Melkonyan, Mo Sun, Rajnikant Patel, Leif W. Ellisen, Tim Robinson, Dong Song, Siang-Boon Koh

**Affiliations:** 1 University of Bristol, University Walk, Bristol, UK; 2 College of Pharmacy, Al-Kitab University, Kirkuk, Iraq; 3 Department of Breast Surgery, General Surgery Centre, The First Hospital of Jilin University, Changchun, Jilin China; 4 Massachusetts General Hospital, Boston, MA USA; 5 Department of Pathology, The First Hospital of Jilin University, Changchun, Jilin China; 6 University of Leicester, Henry Wellcome Building, Lancaster, UK; 7 Harvard Medical School, Boston, MA USA; 8 University Hospitals Bristol and Weston, NHS Foundation Trust, Bristol, UK

**Keywords:** Oncogenes, Breast cancer, Apoptosis

Correction to: *Oncogene* 10.1038/s41388-025-03284-5, published online 31 January 2025

In the Acknowledgements section of this article the grant number relating to University of Bristol was incorrectly given as (R101922-123) and should have been ([222064/Z/20/Z]).

The original article has been corrected.

