# Supplementary material for: CREB1-BCL2 drives mitochondrial resilience in RAS GAP-dependent breast cancer chemoresistance
Source: Oncogene. 2025 Jan 31;44(16):1093–105. doi: 10.1038/s41388-025-03284-5 (PMC11996675; doi:10.1038/s41388-025-03284-5)
Supplement: Supplementary file 1 — Supplementary figures [file 41388_2025_3284_MOESM1_ESM.pptx]

## Slide 1
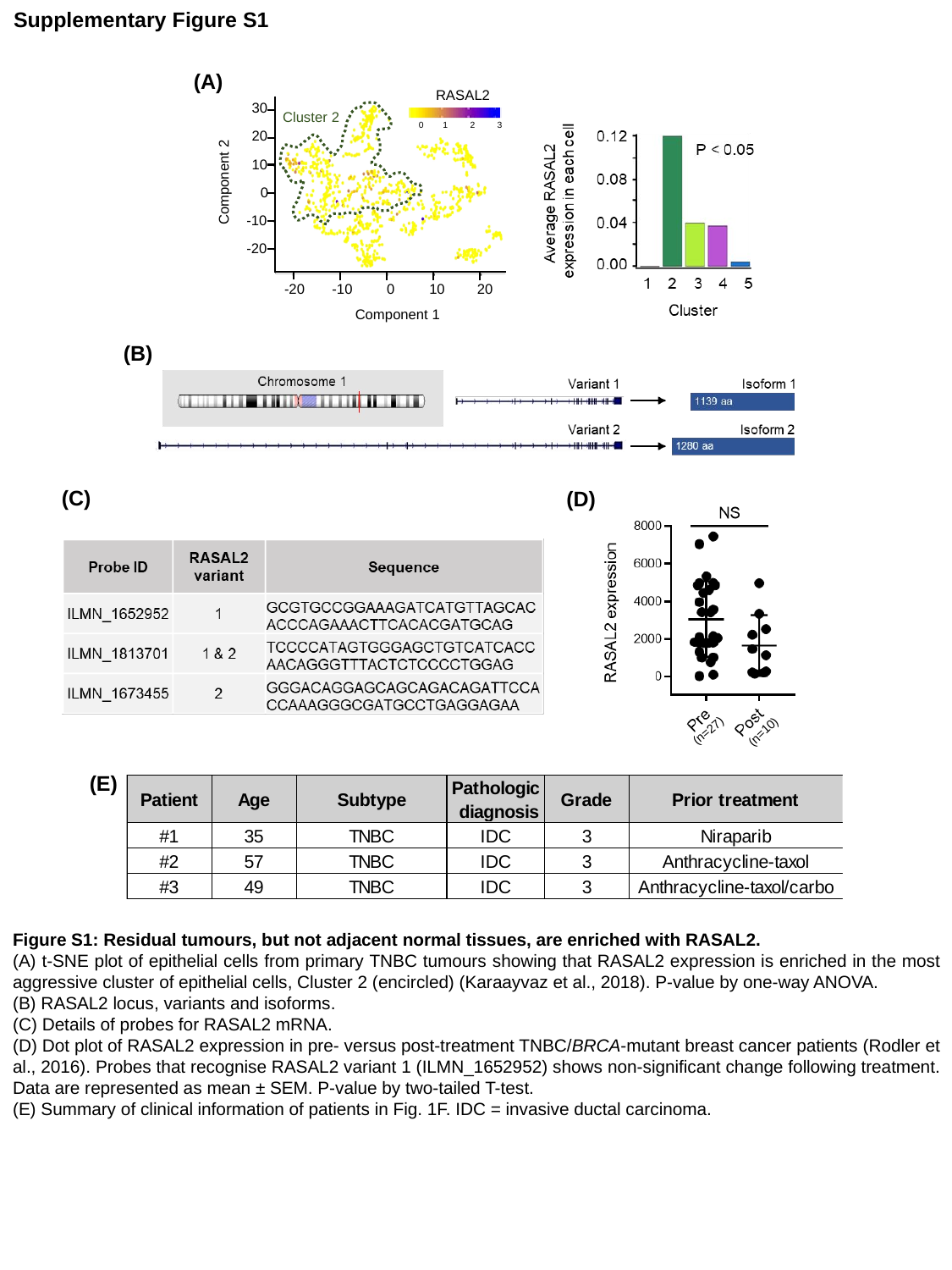

Supplementary Figure S1
(A)
RASAL2
30
Cluster 2
0
1
2
3
20
10
Component 2
0
-10
-20
-20
-10
0
10
20
Component 1
(B)
(C)
(D)
(n=27)
(n=10)
(E)
Figure S1: Residual tumours, but not adjacent normal tissues, are enriched with RASAL2.
(A) t-SNE plot of epithelial cells from primary TNBC tumours showing that RASAL2 expression is enriched in the most aggressive cluster of epithelial cells, Cluster 2 (encircled) (Karaayvaz et al., 2018). P-value by one-way ANOVA.
(B) RASAL2 locus, variants and isoforms.
(C) Details of probes for RASAL2 mRNA.
(D) Dot plot of RASAL2 expression in pre- versus post-treatment TNBC/BRCA-mutant breast cancer patients (Rodler et al., 2016). Probes that recognise RASAL2 variant 1 (ILMN_1652952) shows non-significant change following treatment. Data are represented as mean ± SEM. P-value by two-tailed T-test.
(E) Summary of clinical information of patients in Fig. 1F. IDC = invasive ductal carcinoma.

## Slide 2
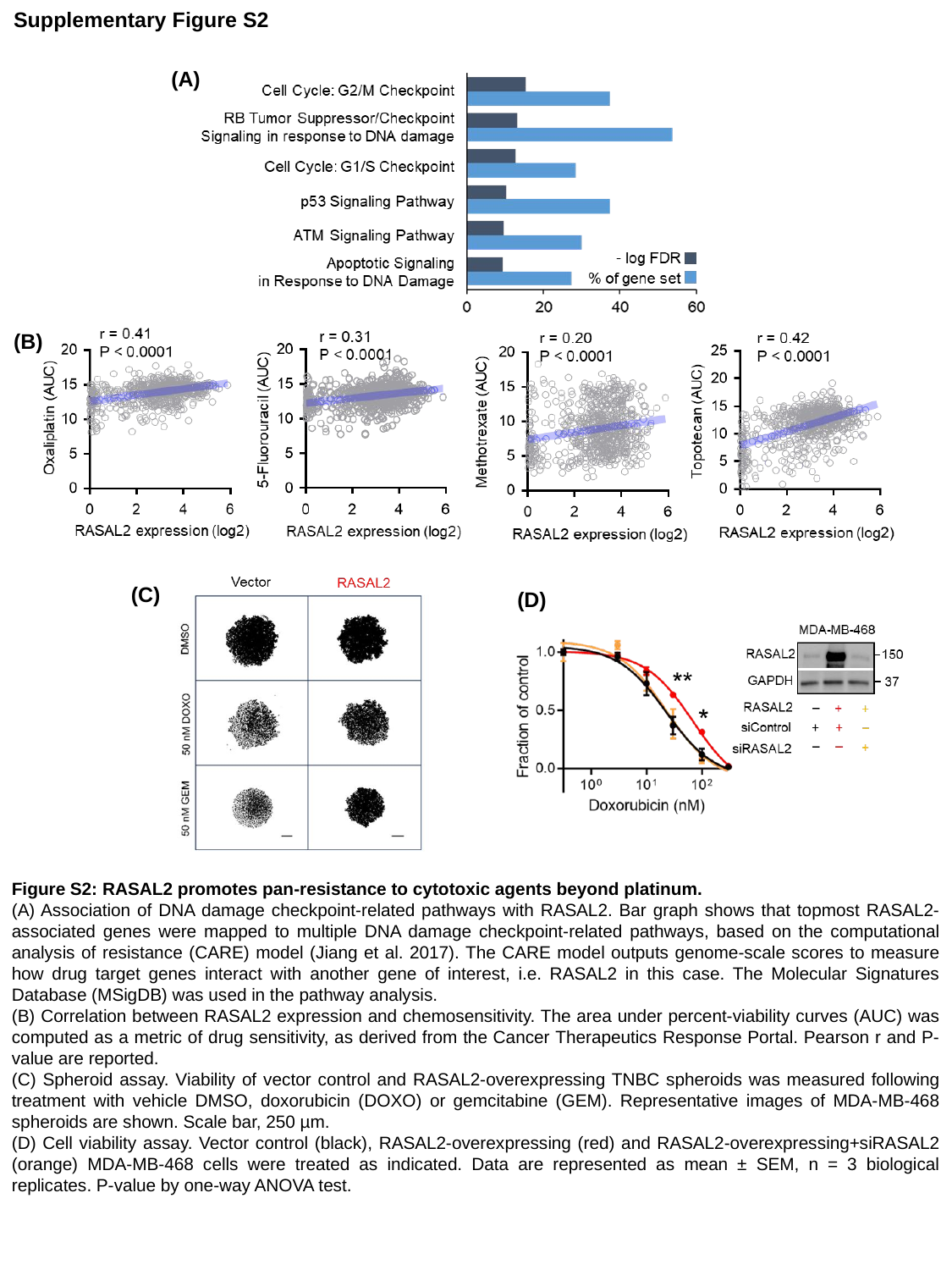

Supplementary Figure S2
(A)
(B)
(C)
(D)
Figure S2: RASAL2 promotes pan-resistance to cytotoxic agents beyond platinum.
(A) Association of DNA damage checkpoint-related pathways with RASAL2. Bar graph shows that topmost RASAL2-associated genes were mapped to multiple DNA damage checkpoint-related pathways, based on the computational analysis of resistance (CARE) model (Jiang et al. 2017). The CARE model outputs genome-scale scores to measure how drug target genes interact with another gene of interest, i.e. RASAL2 in this case. The Molecular Signatures Database (MSigDB) was used in the pathway analysis.
(B) Correlation between RASAL2 expression and chemosensitivity. The area under percent-viability curves (AUC) was computed as a metric of drug sensitivity, as derived from the Cancer Therapeutics Response Portal. Pearson r and P-value are reported.
(C) Spheroid assay. Viability of vector control and RASAL2-overexpressing TNBC spheroids was measured following treatment with vehicle DMSO, doxorubicin (DOXO) or gemcitabine (GEM). Representative images of MDA-MB-468 spheroids are shown. Scale bar, 250 µm.
(D) Cell viability assay. Vector control (black), RASAL2-overexpressing (red) and RASAL2-overexpressing+siRASAL2 (orange) MDA-MB-468 cells were treated as indicated. Data are represented as mean ± SEM, n = 3 biological replicates. P-value by one-way ANOVA test.

## Slide 3
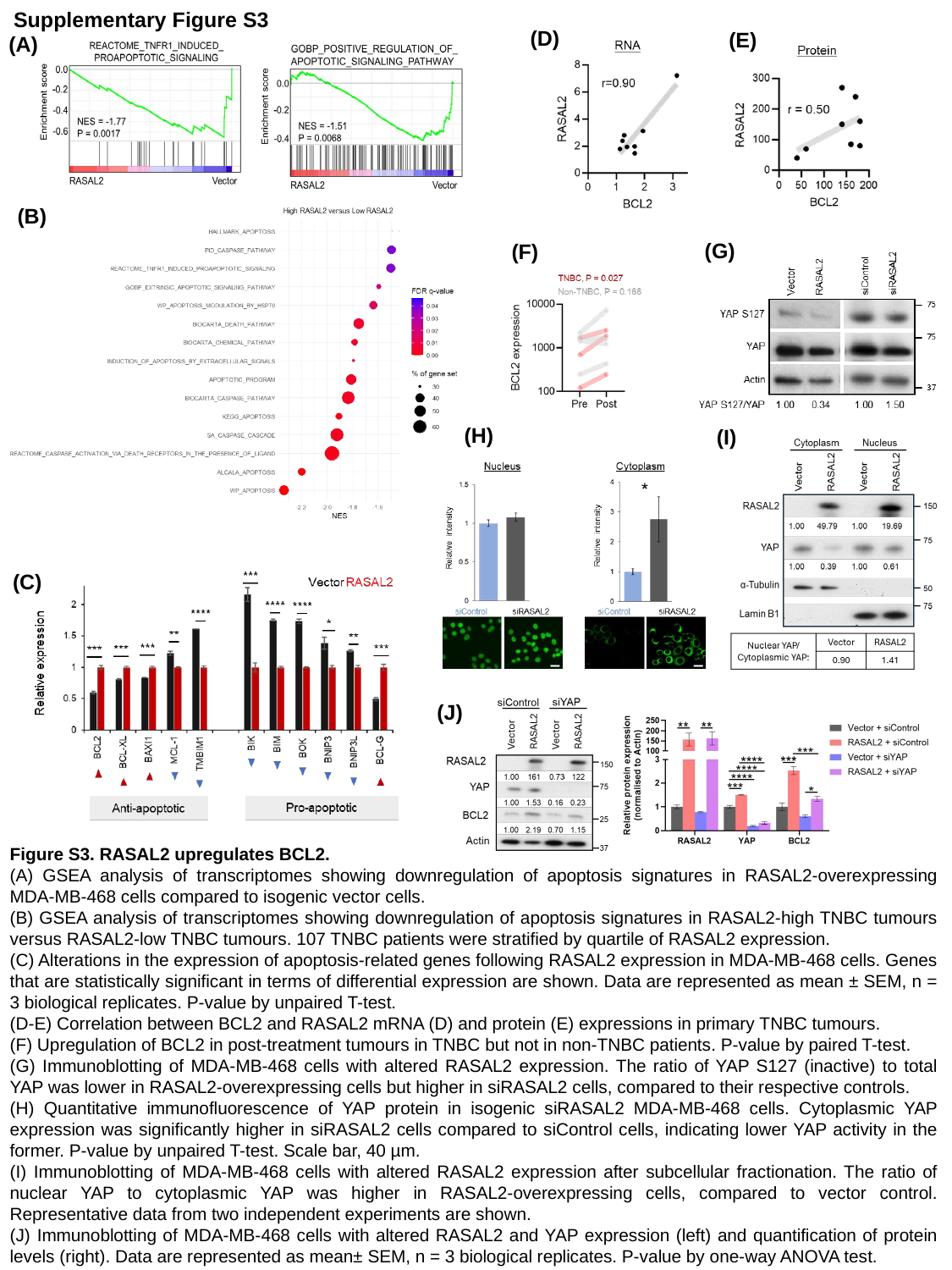

Supplementary Figure S3
(D)
(E)
(A)
(B)
(G)
(F)
(H)
(I)
(C)
(J)
Figure S3. RASAL2 upregulates BCL2.
(A) GSEA analysis of transcriptomes showing downregulation of apoptosis signatures in RASAL2-overexpressing MDA-MB-468 cells compared to isogenic vector cells.
(B) GSEA analysis of transcriptomes showing downregulation of apoptosis signatures in RASAL2-high TNBC tumours versus RASAL2-low TNBC tumours. 107 TNBC patients were stratified by quartile of RASAL2 expression.
(C) Alterations in the expression of apoptosis-related genes following RASAL2 expression in MDA-MB-468 cells. Genes that are statistically significant in terms of differential expression are shown. Data are represented as mean ± SEM, n = 3 biological replicates. P-value by unpaired T-test.
(D-E) Correlation between BCL2 and RASAL2 mRNA (D) and protein (E) expressions in primary TNBC tumours.
(F) Upregulation of BCL2 in post-treatment tumours in TNBC but not in non-TNBC patients. P-value by paired T-test.
(G) Immunoblotting of MDA-MB-468 cells with altered RASAL2 expression. The ratio of YAP S127 (inactive) to total YAP was lower in RASAL2-overexpressing cells but higher in siRASAL2 cells, compared to their respective controls.​
(H) Quantitative immunofluorescence of YAP protein in isogenic siRASAL2 MDA-MB-468 cells. Cytoplasmic YAP expression was significantly higher in siRASAL2 cells compared to siControl cells, indicating lower YAP activity in the former. P-value by unpaired T-test. Scale bar, 40 µm.
(I) Immunoblotting of MDA-MB-468 cells with altered RASAL2 expression after subcellular fractionation. The ratio of nuclear YAP to cytoplasmic YAP was higher in RASAL2-overexpressing cells, compared to vector control. Representative data from two independent experiments are shown.
(J) Immunoblotting of MDA-MB-468 cells with altered RASAL2 and YAP expression (left) and quantification of protein levels (right). Data are represented as mean± SEM, n = 3 biological replicates. P-value by one-way ANOVA test.

## Slide 4
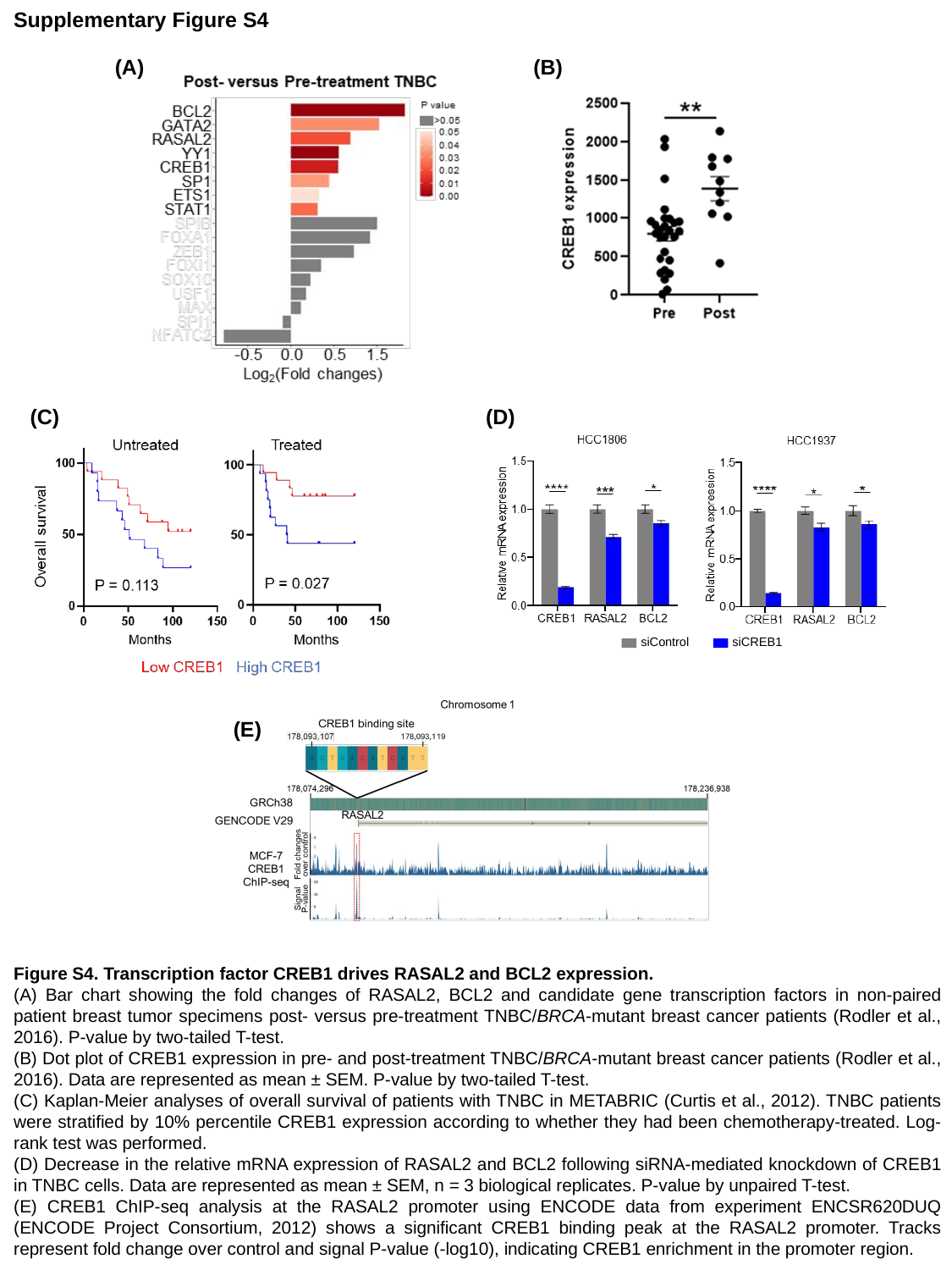

Supplementary Figure S4
(A)
(B)
(C)
(D)
siControl
siCREB1
(E)
Figure S4. Transcription factor CREB1 drives RASAL2 and BCL2 expression.
(A) Bar chart showing the fold changes of RASAL2, BCL2 and candidate gene transcription factors in non-paired patient breast tumor specimens post- versus pre-treatment TNBC/BRCA-mutant breast cancer patients (Rodler et al., 2016). P-value by two-tailed T-test.
(B) Dot plot of CREB1 expression in pre- and post-treatment TNBC/BRCA-mutant breast cancer patients (Rodler et al., 2016). Data are represented as mean ± SEM. P-value by two-tailed T-test.
(C) Kaplan-Meier analyses of overall survival of patients with TNBC in METABRIC (Curtis et al., 2012). TNBC patients were stratified by 10% percentile CREB1 expression according to whether they had been chemotherapy-treated. Log-rank test was performed.
(D) Decrease in the relative mRNA expression of RASAL2 and BCL2 following siRNA-mediated knockdown of CREB1 in TNBC cells. Data are represented as mean ± SEM, n = 3 biological replicates. P-value by unpaired T-test.
(E) CREB1 ChIP-seq analysis at the RASAL2 promoter using ENCODE data from experiment ENCSR620DUQ (ENCODE Project Consortium, 2012) shows a significant CREB1 binding peak at the RASAL2 promoter. Tracks represent fold change over control and signal P-value (-log10), indicating CREB1 enrichment in the promoter region.

## Slide 5
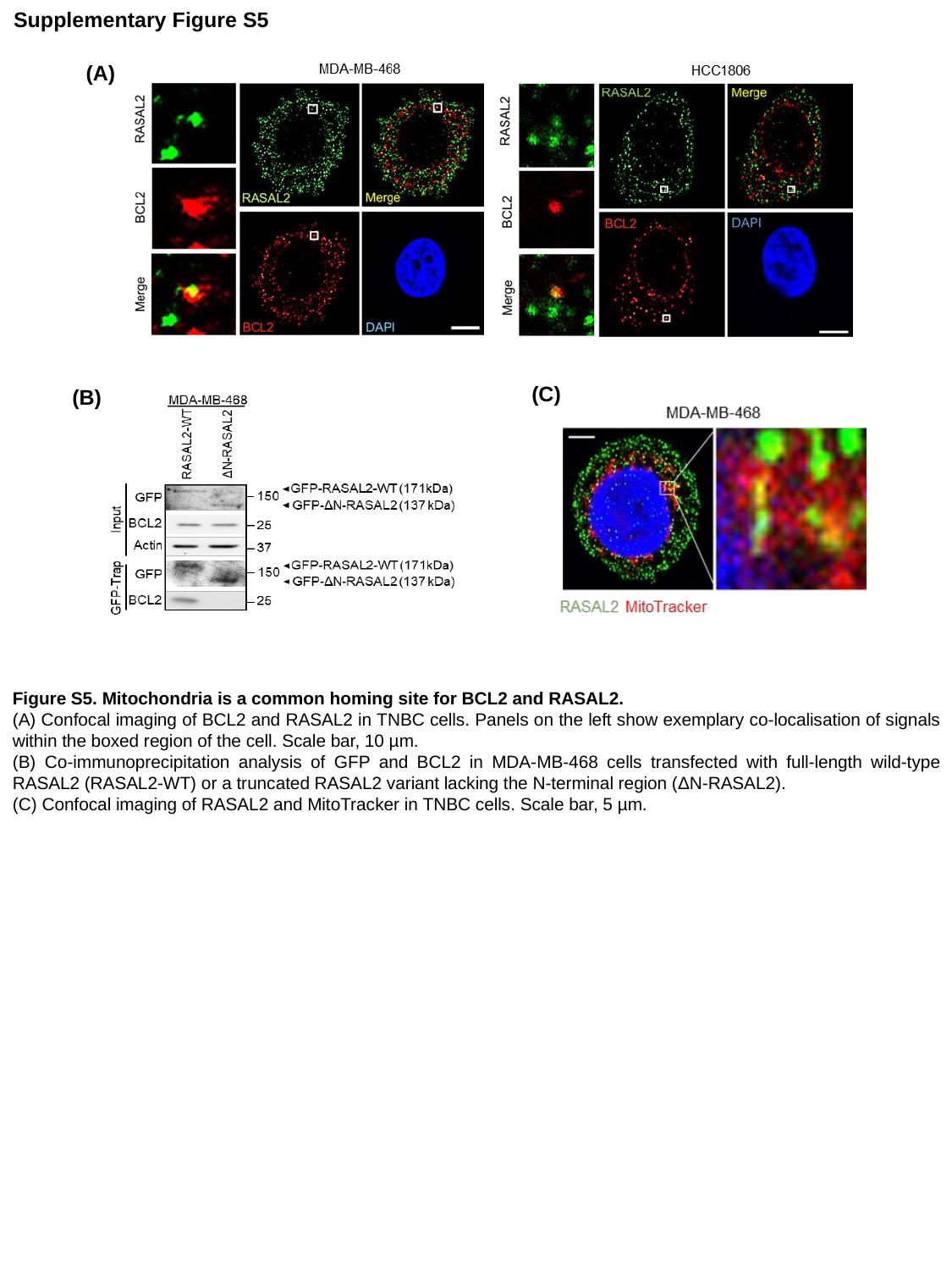

Supplementary Figure S5
(A)
(C)
(B)
Figure S5. Mitochondria is a common homing site for BCL2 and RASAL2.
(A) Confocal imaging of BCL2 and RASAL2 in TNBC cells. Panels on the left show exemplary co-localisation of signals within the boxed region of the cell. Scale bar, 10 µm.
(B) Co-immunoprecipitation analysis of GFP and BCL2 in MDA-MB-468 cells transfected with full-length wild-type RASAL2 (RASAL2-WT) or a truncated RASAL2 variant lacking the N-terminal region (ΔN-RASAL2).
(C) Confocal imaging of RASAL2 and MitoTracker in TNBC cells. Scale bar, 5 µm.

## Slide 6
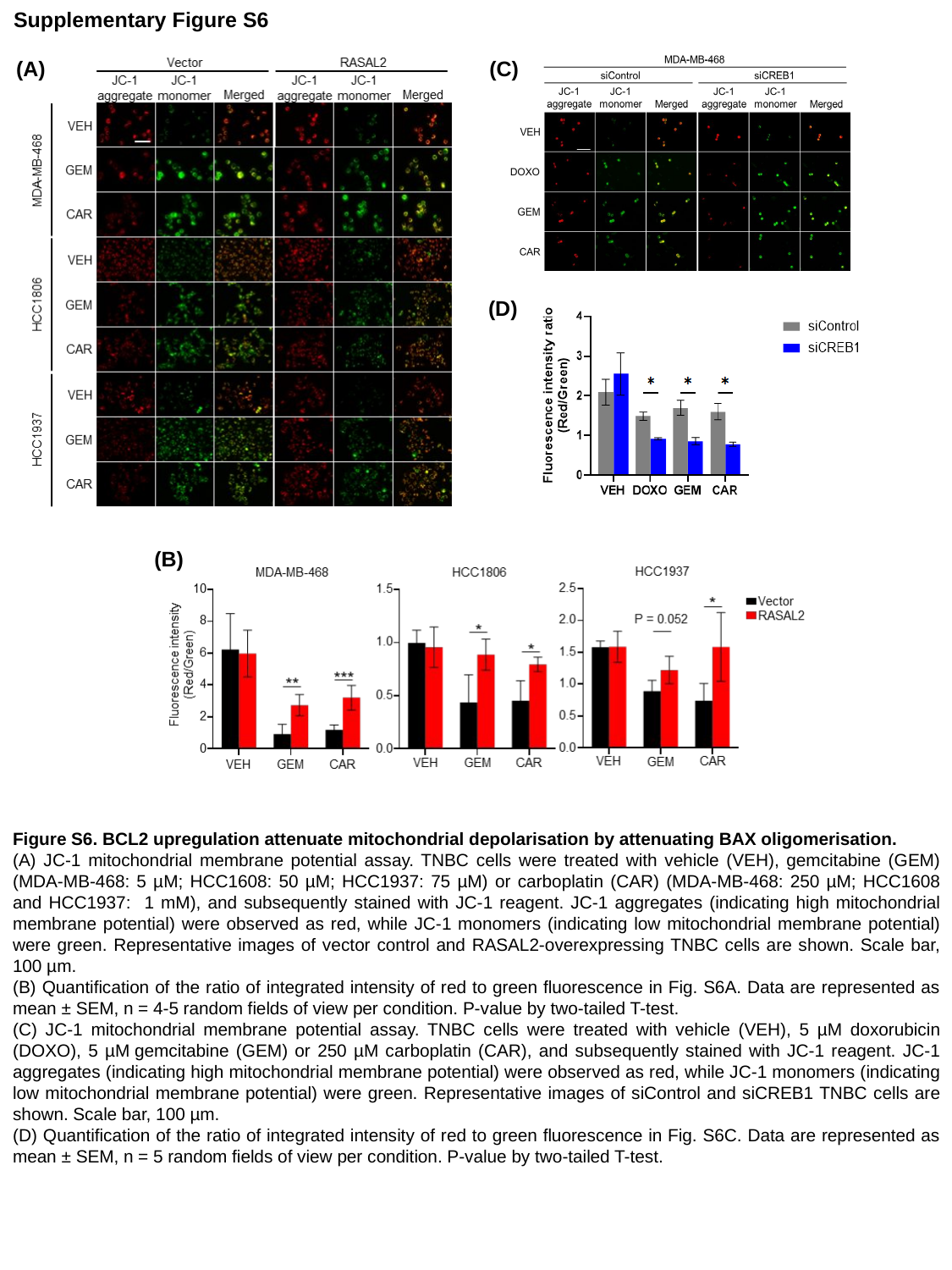

Supplementary Figure S6
(A)
(C)
(D)
(B)
Figure S6. BCL2 upregulation attenuate mitochondrial depolarisation by attenuating BAX oligomerisation.
(A) JC-1 mitochondrial membrane potential assay. TNBC cells were treated with vehicle (VEH), gemcitabine (GEM) (MDA-MB-468: 5 µM; HCC1608: 50 µM; HCC1937: 75 µM) or carboplatin (CAR) (MDA-MB-468: 250 µM; HCC1608 and HCC1937: 1 mM), and subsequently stained with JC-1 reagent. JC-1 aggregates (indicating high mitochondrial membrane potential) were observed as red, while JC-1 monomers (indicating low mitochondrial membrane potential) were green. Representative images of vector control and RASAL2-overexpressing TNBC cells are shown. Scale bar, 100 µm.
(B) Quantification of the ratio of integrated intensity of red to green fluorescence in Fig. S6A. Data are represented as mean ± SEM, n = 4-5 random fields of view per condition. P-value by two-tailed T-test. ​
(C) JC-1 mitochondrial membrane potential assay. TNBC cells were treated with vehicle (VEH), 5 µM doxorubicin (DOXO), 5 µM gemcitabine (GEM) or 250 µM carboplatin (CAR), and subsequently stained with JC-1 reagent. JC-1 aggregates (indicating high mitochondrial membrane potential) were observed as red, while JC-1 monomers (indicating low mitochondrial membrane potential) were green. Representative images of siControl and siCREB1 TNBC cells are shown. Scale bar, 100 µm.
(D) Quantification of the ratio of integrated intensity of red to green fluorescence in Fig. S6C. Data are represented as mean ± SEM, n = 5 random fields of view per condition. P-value by two-tailed T-test.
