# Supplementary material for: CREB1-BCL2 drives mitochondrial resilience in RAS GAP-dependent breast cancer chemoresistance
Source: Oncogene. 2025 Jan 31;44(16):1093–105. doi: 10.1038/s41388-025-03284-5 (PMC11996675; doi:10.1038/s41388-025-03284-5)
Supplement: Supplementary file 2 — Supplementary tables [file 41388_2025_3284_MOESM2_ESM.docx]

**Supplementary Table 1.** Sequence of primers used.

| **Primer Name** | **Sequence (5’ - 3’)** |
| --- | --- |
| BCL2-F | GGTGGGGTCATGTGTGTGG |
| BCL2-R | CGGTTCAGGTACTCAGTCATCC |
| CREB1-F | ATTCACAGGAGTCAGTGGATAGT |
| CREB1-R | CACCGTTACAGTGGTGATGG |
| RASAL2-F | ACGTGGGAGCGGAAGTATTG |
| RASAL2-R | TGTCCCCCTTCCACAGGTAT |
| β-ACTIN-F | CATCCACGAAACTACCTTCAACTC |
| β-ACTIN-R | GAGCCGCCGATCCACACG |

| **Antibody** | **Manufacturer** | **Catalogue number** | **Application**  **(WB = Western blotting, IP = immunoprecipitation)** | **Dilution factor** |
| --- | --- | --- | --- | --- |
| AKT/PKB | Millipore | 05-591-25UG | WB | 1-1000 |
| BAX | Cell Signaling | 2772 | WB | 1-1000 |
| BCL2 | Dako | M0887 | WB | 1-1000 |
| BCL2 | Cell Signaling | 3498 | WB | 1-1000 |
| BCL2 (C-2) | Santa Cruz Biotechnology | sc-7382 | IP | 2µg per 1000 µg of total protein |
| Beta Actin | Proteintech | 66009-1-Ig | WB | 1-10000 |
| Cleaved Caspase-3 | Cell Signaling | 9664 | WB | 1-1000 |
| CREB1 | Cell Signaling | 9197 | WB, Chromatin IP | 1-1000; 1-50 |
| Cytochrome C | BD Pharmingen | 556433 | WB | 1-1000 |
| GAPDH | Santa Cruz Biotechnology | sc-25778 | WB | 1-1000 |
| Lamin B1 | Proteintech | 12987-1-AP | WB | 1-20000 |
| Normal Rabbit IgG | Merck | 12-370 | Chromatin IP | 1.14µg in 500µL |
| Phospho-YAP (Ser127) | Cell Signaling | 4911 | WB | 1-1000 |
| RASAL2 (B-11) | Santa Cruz Biotechnology | sc-390605 | IP | 2µg per 1000 µg of total protein |
| RASAL2 (D6K9L) | Cell Signaling | 82481 | WB | 1-2000 |
| TOM20 | ABclonal | A19403 | WB | 1-1000 |
| YAP (D8H1X) | Cell Signaling | 14074 | WB | 1-1000 |
| yH2AX | Millipore | Merck 05-636 | WB | 1-1000 |
| Alpha Tubulin | Santa Cruz Biotechnology | sc-32293 | WB | 1-2000 |
| GFP | Novus Biologicals | NB100-1614 | WB | 1-5000 |

**Supplementary Table 2.** Details of antibodies for western blotting and immunoprecipitation

**Supplementary Table 3.** Details of antibodies for immunofluorescence and immunohistochemistry

| **Antibody** | **Manufacturer** | **Catalogue number** | **Application**  **(IF = immunofluorescence, IHC = immunohistochemistry)** | **Dilution factor** |
| --- | --- | --- | --- | --- |
| BCL2 | Dako | M0887 | IF | 1-100 |
| BCL2 | Proteintech | 68103-1-Ig | IHC | 1-1000 |
| RASAL2 | ThermoFisher | PA5-31791 | IHC | 1-1000 |
| RASAL2 (D6K9L) | Cell Signaling | 82481 | IF | 1-100 |
| YAP (D8H1X) | Cell Signaling | 14074 | IF | 1-100 |
